# Supplementary material for: Predicting visual function by interpreting a neuronal wiring diagram
Source: Nature. 2024 Oct 2;634(8032):113–23. doi: 10.1038/s41586-024-07953-5 (PMC11446822; doi:10.1038/s41586-024-07953-5)
Supplement: Supplementary file 6 — CRF and ERF predictions for individual TmY4 and TmY9 cells. Analogous to Supplementary Data 3, but for TmY target types. Shown are the top four monosynaptic pathways, the strongest pathway passing through each of the top ten intermediary types (ranking from Extended Data Fig. 7), and the trisynaptic pathway Tm1–TmY–Dm3–TmY (see the section entitled Prediction of spatial normalization). [file 41586_2024_7953_MOESM6_ESM.zip › DataS4/TmY9qΓèÑ/720575940612276066.pdf]

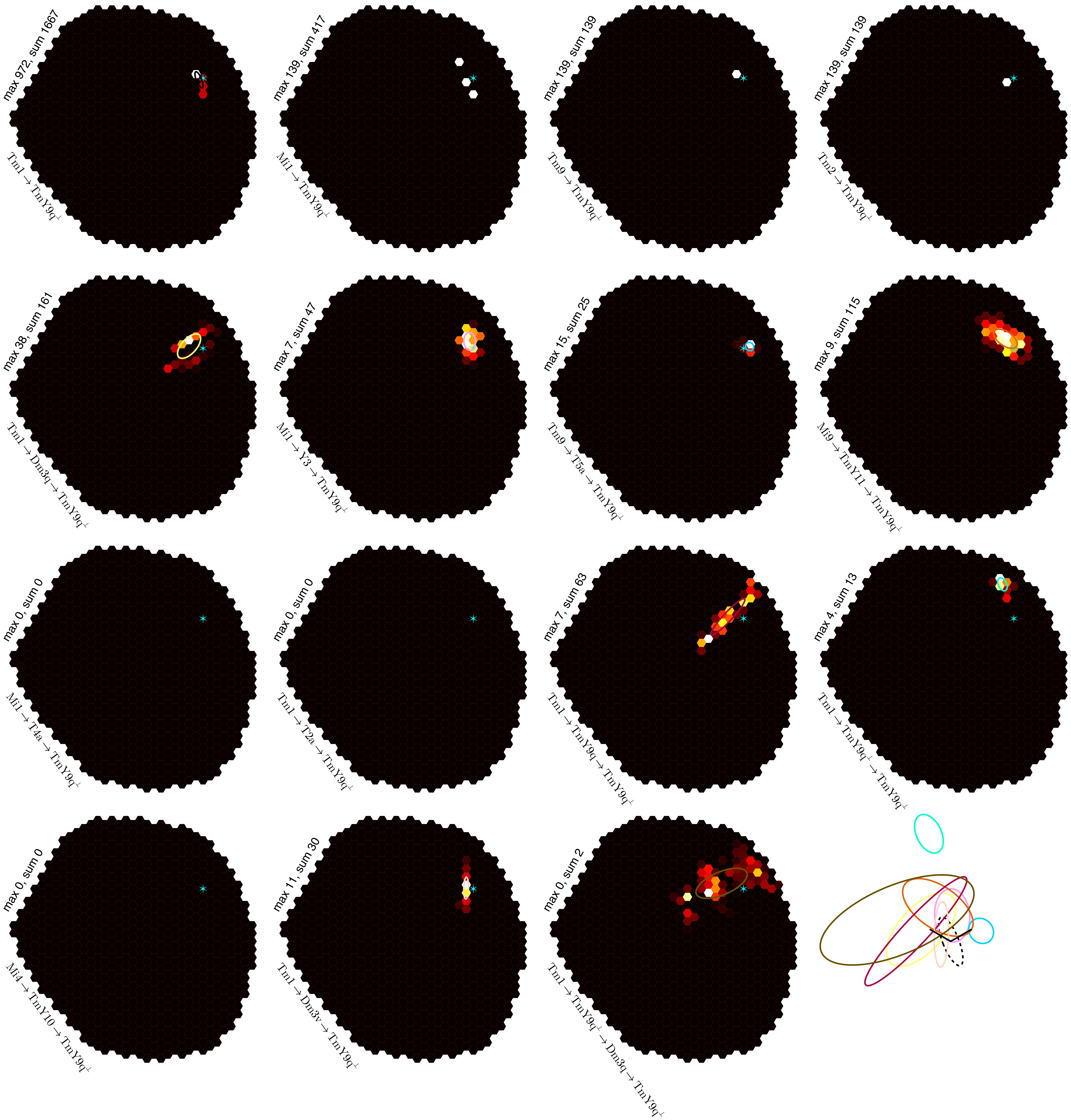
max 972, sum 1667
Tm1 → TmY9q⊥
max 139, sum 417
Mi1 → TmY9q⊥
max 139, sum 139
Tm9 → TmY9q⊥
max 139, sum 139
Tm2 → TmY9q⊥
max 38, sum 161
Tm1 → Dm3q → TmY9q⊥
max 7, sum 47
Mi1 → Y3 → TmY9q⊥
max 15, sum 25
Tm9 → T5a → TmY9q⊥
max 9, sum 115
Mi9 → TmY11 → TmY9q⊥
max 0, sum 0
Mi1 → T4a → TmY9q⊥
max 0, sum 0
Tm1 → T2a → TmY9q⊥
max 7, sum 63
Tm1 → TmY9q → TmY9q⊥
max 4, sum 13
Tm1 → TmY9q⊥ → TmY9q⊥
max 0, sum 0
Mi4 → TmY10 → TmY9q⊥
max 11, sum 30
Tm1 → Dm3v → TmY9q⊥
max 0, sum 2
Tm1 → TmY9q⊥ → Dm3q → TmY9q⊥
